# Pain Management and Sociology Implications: The Sociomedical Problem of Pain Clinic Staff Harassment Caused by Chronic Pain Patients

**DOI:** 10.5812/aapm-144263

**Published:** 2024-05-05

**Authors:** Olumuyiwa Akinwumi Bamgbade, Monisola Temidayo Sonaike, Leili Adineh-Mehr, Daniel Olutosin Bamgbade, Zaina Samir Aloul, Cherith Boatametse Thanke, Thakgalo Thibela, Grace Gaceri Gitonga, Genet Tadesse Yimam, Aria Genaelle Mwizero, Fidelia Batombari Alawa, Lahja Omagano Kamati, Nolubabalo Patience Ralasi, Mwewa Chansa

**Affiliations:** 1 University of British Columbia, Vancouver, Canada; 2 Salem Anaesthesia Pain Clinic, Surrey, Canada; 3 University College Hospital, Ibadan, Nigeria; 4 Iranian Pain Society, Tehran, Iran; 5 University of Cardiff , Cardiff, United Kingdom; 6 University of Botswana, Gaborone, Botswana; 7 University of the Witwatersrand, Johannesburg, South Africa; 8 University of Nairobi, Nairobi, Kenya; 9 United Nations Economic Commission for Africa, Addis Ababa, Ethiopia; 10 University of Rwanda, Kigali, Rwanda; 11 University of Port Harcourt, Port Harcourt, Nigeria; 12 University of Namibia, Windhoek, Namibia; 13 Department of Eastern Cape Health, Port Elizabeth, South Africa; 14 University Teaching Hospital, Lusaka, Zambia

**Keywords:** Chronic Pain, Medical Sociology, Workplace Harassment, Protection of Healthcare Workers, Healthcare Racism, Sexual Harassment, Retaliation Harassment, Opioid Dependency, Sedative Dependency

## Abstract

**Background:**

Patients with chronic pain often experience psychological issues. They may also exhibit harassing behaviors toward healthcare staff. This complex sociomedical issue necessitates increased attention.

**Objectives:**

This study analyzed incidents of staff harassment caused by chronic pain patients. It examined the characteristics of chronic pain patients who harassed clinic staff, as well as the causative or associated factors. The study also explored the management and outcomes of these harassment incidents.

**Methods:**

This prospective observational study involved 1102 chronic pain patients who received treatment at a pain clinic. Data were prospectively collected on patients' gender, age, ethnicity, occupation, injury insurance claims, and incidents of staff harassment caused by patients.

**Results:**

Pain clinic staff were harassed by 121 patients (11%). Among the harassers, females constituted 70.2% and males 29.8%. Additionally, 50.4% of the harassers were unemployed, with unemployed patients causing more staff harassments (P = 0.001). A significant portion, 86 %, of the harassers had injury insurance claims and were associated with a higher incidence of staff harassments (P = 0.002). Patients making disability insurance claims also caused more staff harassments (P = 0.001). Among the harassers, 50.4 % demanded higher drug doses, and 50% did not have regular primary healthcare providers. The types of harassment included insults (34.7%), threats (19.8%), retaliations (3.3%), and sexual harassment (42.2%). All cases of sexual harassment were addressed; the patients involved were counseled. Most harassment incidents were resolved through tactful communication. Of the harassers, 9.9 % were discharged from the clinic.

**Conclusions:**

Harassment of pain clinic staff by chronic pain patients is significant. This sociomedical issue may be worsening due to factors such as opioid misuse, racism, the pandemic, and socioeconomic challenges. While most chronic pain patients are reasonable, some can be challenging. This study confirmed that the majority of patients who harassed staff were female, unemployed, had made injury insurance claims, and demanded higher drug doses. Abusive patients should receive anxiolytic therapy, behavioral boundaries, counseling, distraction therapy, and empathy. Pain clinics should implement staff training and support programs to protect staff from harassment. Additionally, pain clinicians should establish peer support networks to mitigate the psychological impacts of patient aggression and maintain professional well-being.

## 1. Background

Chronic pain is associated with psychosomatic comorbidities (1-4). Patients with chronic pain typically experience associated problems such as insomnia, drug dependence, anxiety, distress, confusion, and other psychological disorders (3-7). Clinicians often find it challenging to discuss the reduction of opioids or sedatives with these patients, as many drug-dependent patients respond negatively to such necessary treatment adjustments (4-6). Many chronic pain patients are dissatisfied with their analgesic or anxiolytic dosages, and some harass or threaten healthcare providers (4, 6). These patients frequently demand higher doses of opioids, zopiclone, benzodiazepines, and other mood-altering medications. Occasionally, some of these patients engage in significant harassment, threats, or violence toward clinical staff (6, 8). Most pain care providers fail to report or address the harassment caused by patients and their families (6, 9). Additionally, many healthcare staff lack the empowerment to manage or mitigate harassment from patients and their families (10-13).

Various factors may contribute to patients' negative behavior towards pain care providers. Hostility from patients towards pain clinicians may stem from psychological, physical, pathological, and social issues. Such negative behaviors can also arise from patients' anxiety, pain, insomnia, drug addiction, and drug withdrawal syndrome. Harassment of healthcare staff by chronic pain patients may be linked to feelings of hopelessness, powerlessness, helplessness, restlessness, and loneliness. Harassment of pain clinic staff by patients and their families represents a significant sociomedical challenge. However, there is a lack of adequate information, recommendations, and actions addressing this healthcare issue. Additionally, there are no previous major clinical studies specifically focusing on actual incidents of harassment towards pain clinic staff.

## 2. Objectives

This clinical research analyzed the issue of harassment of pain clinic staff caused by chronic pain patients. It examined the prevalence, types, and incidents of clinic staff harassment. The study also investigated the demographics and characteristics of chronic pain patients who harassed the clinic staff. Additionally, it analyzed the incidents of harassment, along with the causative or associated factors. Finally, the study explored the management and outcomes of these harassment incidents.

## 3. Methods

### 3.1. Study Design

This is a prospective observational cohort clinical study involving consecutive patients. The clinical research protocol was registered on the Clinical Trials PRS (Protocol Registration and Results System) website with the PRS number NCT05876104. Conducted at an interventional pain clinic in Canada, this study focuses on routine clinical care. It serves as a quality assurance investigation of routine clinical practice and social behavior. Approval was granted by the healthcare organization and the pain clinic. The study encompassed adult patients who received pain management at the specialist pain clinic. The clinic was staffed by four professionals: Two male Black professionals, one female Black professional, and one female Asian professional.

The study evaluated documented incidents of harassment toward pain clinic staff, caused by patients and their families. It analyzed causative factors of the incidents, associated patient factors, outcomes of the incidents, system-related factors, and lessons learned. This research involved a quantitative analysis of data from prospective electronic records and incident diaries collected during routine clinical care. The risk of patient selection bias was minimized through the use of consecutive sampling. Additionally, the risks of recall bias and outcome bias were reduced by the prospective nature of the data collection. The cohort methodology facilitated the gathering of data on multiple variables and outcomes throughout the specific study duration, potentially revealing new associations between variables and outcomes.

### 3.2. Data Collection

This observational study involved 1102 consecutive patients who underwent pain management from January 2018 to December 2023. This convenience sample included all treated patients. Clinical data collection encompassed patients’ gender, age, ethnicity, occupation, type of injury insurance claim, and incidents of staff harassment by patients or their families. Gender was categorized as male, female, or transgender. Age groups were defined as 17 - 30 years (youth), 31 - 45 years (early adult), 46 - 64 years (middle-aged), and 65 - 99 years (elderly). Ethnicity was categorized into several groups: Black, Indigenous (First Nations, Metis), White, Central Asian (Afghanistan, Iran, Kazakhstan), Arab, Southeast Asian (Philippines, Vietnam, Cambodia, Thailand), Latino, Northeast Asian (China, Japan, Korea), and South Asian (India, Pakistan, Bangladesh, Nepal). Occupations were classified as Nurse/Doctor, Police, Office work, Skilled work, Unskilled work, Retired, and Unemployed. Types of injury insurance claims included work injury, disability injury, car accident injury, and fall injury.

Incidents of clinic staff harassment caused by patients were categorized into insults, threats, retaliation complaints, and sexual harassment. Insults from patients varied and included racist, xenophobic, derogatory, name-calling, and sexist abuse. Threats from patients encompassed complaints to authorities, negative customer reviews, vengeance, vandalism, and physical violence. Retaliation complaint harassment involved patients making spurious complaints to authorities and lodging unfair medicolegal complaints. Types of sexual harassment included propositions of intimate or sexual activity, comments about staff's anatomy or body, unwanted sexual remarks, unwanted hugging or touching, repeated social or date invitations, unsolicited descriptions by patients of their sexual activities, and patients soliciting comments about their own body or appearance.

### 3.3. Statistical Methods

Data were analyzed using IBM® SPSS® Statistics 28 (IBM Corp, Armonk, NY) through methods such as Student's *t*-test, analysis of variance, regression analysis, Pearson Chi-square test, and Fisher's exact test. A P-value < 0.05 was considered significant. Both quantitative and qualitative data are presented in tables, which include numbers, categories, descriptions, ranges, and percentages. The data have been compared, analyzed, and interpreted appropriately.

## 4. Results

The study included 1,102 chronic pain patients, consisting of 65% females, 34.6% males, and 0.4% transgender patients. The pain clinic staff comprised four professionals: Two male Black professionals, a female Black professional, and a female Asian professional.

Table 1 presents the patients’ demographics and characteristics. The age distribution was as follows: Youth (6.7%), early adults (24.3%), middle-aged (47.8%), and elderly (21.2%) patients. The ethnicity distribution included Arab (2.6%), Black (3.8%), Central Asian (2.7%), Indigenous (4.8%), Latino (5%), Northeast Asian (4.7%), Southeast Asian (4.5%), South Asian (20.2%), and White (51.7%) patients. The occupational pattern was composed of police officers (1.3%), nurse/doctor professionals (3%), office workers (13.7%), unskilled workers (16.7%), skilled workers (17.8%), retired individuals (22.2%), and unemployed individuals (25.3%). Of the patients, 456 (41.4%) had no legal claims for injury, while 646 (58.6%) made legal claims for injury, including 17 fall injury claims (1.5%), 123 work injury claims (11.2%), 213 disability injury claims (19.3%), and 293 car accident injury claims (26.6%).

**Table 1.** Characteristics of Chronic Pain Patients Who Underwent Treatment at the Pain Clinic During January 2018 to December 2023 (N = 1102)

Parameter	No. (%)
**Sex**	
Female	717 (65)
Male	381 (34.6)
Transgender	4 (0.4)
**Age (y)**	
Youth (17 – 30)	74 (6.7)
Early adult (31 – 45)	268 (24.3)
Middle-aged (46 – 64)	526 (47.8)
Elderly (65 – 99)	234 (21.2)
**Ethnicity**	
Arab	28 (2.6)
Black	42 (3.8)
Central Asian	30 (2.7)
Indigenous	53 (4.8)
Latino	55 (5)
North East Asian	52 (4.7)
South East Asian	50 (4.5)
South Asian	222 (20.2)
White	570 (51.7)
**Occupation**	
Police	14 (1.3)
Nurse/doctor	33 (3)
Office worker	151 (13.7)
Unskilled worker	184 (16.7)
Skilled worker	196 (17.8)
Retired	245 (22.2)
Unemployed	279 (25.3)
**Injury claim**	
Fall injury	17 (1.5)
Work injury	123 (11.2)
Disability injury	213 (19.3)
Car accident injury	293 (26.6)
No injury claim	456 (41.4)

Table 2 displays data on patients who harassed clinic staff. Out of the total patient population, 121 patients, constituting 11%, harassed clinic staff. Among the harassers, 70.2% were female and 29.8% were male. By age, 51.2% of harassers were middle-aged, 25.6% were early adults, 14.9% were elderly, and 8.3% were youths. Ethnically, 74.4% were White, 18.2% South Asian, 5% Southeast Asian, and 2.5% Latino. Regarding occupation, 2.5% of harassers were police officers, 10.7% retired, 11.6% office workers, 11.6% skilled workers, 13.2% unskilled workers, and 50.4% were unemployed. Unemployed patients were significantly more likely to harass staff (P = 0.001). Concerning insurance claims, 43.8% of harassers had made a disability injury claim, 23.1% a car accident injury claim, 14.9% a work injury claim, 4.1% a fall injury claim, and 14.1% had no injury claim. Patients with any insurance injury claim were significantly more likely to harass staff (P = 0.002), especially those with a disability injury claim (odds ratio = 6.97, P = 0.001). Among the harassers, 50.4% demanded higher drug doses or better treatments, 40.5% quicker processing of their injury claims, and 9.1% unrealistic scheduling for pain clinic or radiology appointments. Half of the harassers did not have a regular primary healthcare provider. Additionally, 8.3% of harassers were accompanied by family members who also participated in the harassment of clinic staff.

**Table 2.** Characteristics of Chronic Pain Patients Who Harassed the Pain Clinic Staff During January 2018 to December 2023 (N = 121)

Parameter	No. (%)
**Sex**	
Female	85 (70.2)
Male	36 (29.8)
Transgender	0 (0)
**Age**	
Youth; 17 - 30 Years	10 (8.3)
Early Adult; 31 - 45 Years	31 (25.6)
Middle-aged 46 - 64 Years	62 (51.2)
Elderly; 65 - 99 Years	18 (14.9)
**Ethnicity**	
Arab	0 (0)
Black	0 (0)
Central Asian	0 (0)
Indigenous	0 (0)
North East Asian	0 (0)
Latino	3 (2.5)
South East Asian	6 (4.9)
South Asian	22 (18.2)
White	90 (74.4)
**Occupation**	
Nurse/doctor	0 (0)
Police	3 (2.5)
Office worker	14 (11.6)
Unskilled worker	16 (13.2)
Skilled worker	14 (11.6)
Retired	13 (10.7)
Unemployed	61 (50.4)
**Injury claim**	
Fall injury	5 (4.1)
Work injury	18 (14.9)
Disability injury	53 (43.8)
Car accident injury	28 (23.1)
No injury claim	17 (14.1)
**Excessive demands**	
Higher drug dose or treatment	61 (50.4)
Quicker injury claim process	49 (40.5)
Unrealistic clinic or radiology appointment	11 (9.1)
**Primary healthcare provider**	
General physician doctor	49 (40.5)
Nurse practitioner	12 (9.9)
None: provider died/retired/moved or provider-patient separation/discharge	60 (49.6)

Table-3 details the incidents of harassment. The breakdown of harassment incidents caused by patients includes 42 cases of insults (34.7%), 24 cases of threats (19.8%), 4 cases of retaliation complaint harassment (3.3%), and 51 cases of sexual harassment (42.2%). The type of harassment was significantly associated with gender (P = 0.003), with insults more commonly from male patients and sexual harassment exclusively from female patients. The type of harassment was also significantly associated with the type of patient’s injury claim (P = 0.011); threats were mostly caused by patients making work injury claims, while sexual harassment was predominantly caused by patients making disability injury claims. Furthermore, the type of harassment was significantly associated with the patient’s ethnicity (P = 0.008); insults and threats were primarily from White patients, retaliation complaint harassment was mostly from female South Asian patients, and sexual harassment was mainly by White and South Asian patients.

**Table 3.** Characteristics of Harassments Caused by Chronic Pain Patients During January 2018 to December 2023 (N = 121)

Harassment Type	Population; No. (%)	Outcome After Remediation	Association with Injury Claim	Association with Gender	Association with Ethnicity
**Insults**	42 (34.7)	Resolved 100 %	All types of claim	Male	White
**Threats**	24 (19.8)	Resolved 83 %, discharged 17 %	Work injury claim	Male; female	White
** Retaliation complaint**	4 (3.3)	Discharged 100%	Disability claim	Female	South Asian
**Sexual harassment**	51 (42.2)	Resolved 92 %, discharged 8%	Disability claim	Female	White; South Asian

All incidents of harassment were appropriately managed by the clinic staff to ensure compassionate and ethical outcomes. Incidents involving insults were resolved through diplomatic communication and patient counseling. Most threats (20 out of 24, or 83%) were addressed with tactful dialogue and counseling. Out of the 121 harassers, 12 (9.9%) were discharged from the clinic. Those discharged included four patients (3.3%) who made repeated severe threats, four patients (3.3%) who engaged in severe or repeated sexual harassment, and all four patients (3.3%) involved in retaliation complaint harassment. All advances of sexual harassment made by patients were firmly rejected by the staff, and the patients received counseling on appropriate boundaries. Three female harassers, after being discharged, stalked the pain clinician. Retaliation complaint harassment involved patients making spurious complaints against clinic staff. Three middle-aged female patients, angered by the rejection of their inappropriate treatment demands and sexual advances, made spurious complaints. Similarly, one middle-aged male patient made spurious complaints after his inappropriate opioid demands were rejected.

## 5. Discussion

Healthcare staff harassment by chronic pain patients is a significant issue that demands attention. Our study found that most patients who engaged in harassment did not have regular primary healthcare providers, suggesting that a lack of continuous healthcare provision contributes to negative patient behavior. Often, patients and their families are not at their best during visits to the pain clinic (6, 7, 14, 15). During these visits, patients may experience anger, confusion, pain, anxiety, or the effects of medication, all of which can lead to strained interactions with clinicians (6, 8, 11, 14, 15). Additionally, patients may be accompanied by family members who are fearful and anxious, further complicating the clinic environment. Our study noted that ten patients were joined by their family members in further harassing the staff. Such extreme behavior by patients’ families may be driven by selfish motives, such as financial gain from insurance claims or prescription drug diversion.

Our study revealed that a minority (11%) of patients harassed the staff, causing significant stress and adversely affecting the provision of pain services. Harassment at the pain clinic included insults, threats, hostile behavior, retaliation complaints, and sexual harassment. Insults were particularly hurtful, including racist, xenophobic, derogatory, name-calling, and sexist abuse. Threats were harmful, especially those involving complaints to authorities, vengeance, negative customer reviews, and physical violence. Working under such threats and insults is inhumane and undermines staff morale, performance, and retention (10, 13, 16, 17). This abuse also negatively impacts staff health and interpersonal relationships (9, 10, 12, 16, 17). Harassment of pain clinicians often mirrors broader societal aggression and the personal struggles of patients. Although described in psychological terms as "responsive behavior," particularly if triggered by a condition, it does not excuse harassment. Chronic pain patients often suffer from hopelessness, loneliness, helplessness, and restlessness; however, these are not justifications for harassment. While chronic pain patients may expect pain clinicians to fulfill many roles for various purposes at different times, it is essential that patients and their families treat pain clinic staff with decency, as clinicians endeavor to provide comprehensive care.

Our study adds to the medical knowledge base. Previous research has shown that male pain clinicians are more likely to experience abuse and threats from patients (6, 8). Among specialists, anesthesiology pain clinicians face the most threats (6, 8). Currently, there are no adequate measures to protect healthcare staff, support victimized staff, or penalize abusive patients. Most pain clinicians do not report harassment from patients (6, 8, 11). Despite these challenges, pain clinicians maintain compassion for their patients' well-being and strive to avoid patient abandonment. They often fear retaliation complaints, negative publicity, contempt from authorities, unfair punishments, and societal indifference. Pain clinic staff also worry about not being believed, that patients will not face penalties, and that no actions will be taken to protect them from further harassment. Our study found that four patients made unfounded retaliation complaints, and three patients stalked the pain clinician after being discharged from the clinic.

Anesthesiologists, pain clinicians, and other healthcare professionals should receive training and support in managing difficult or abusive patients. This training will enhance resilience, coping skills, and self-care among healthcare providers who experience harassment. Anesthesiologists and pain clinicians should have access to resources, education, and peer support networks to mitigate the psychological impact of patient aggression, maintain professional well-being, and ensure career longevity.

Our study revealed that most patients who harassed staff were demanding higher drug doses or better treatments, aligning with previous research that identified opioid and sedative demands as the primary context for staff harassment by chronic pain patients (6, 8, 11). Approximately one-tenth of the harassers in our study demanded unrealistic clinical appointments, possibly reflecting their anxiety. Additionally, 40% of the harassers demanded quicker processing of their injury claims, a figure significantly higher than previously reported, underscoring the growing influence of socioeconomic factors on the problem of staff harassment by chronic pain patients (6, 8). This is consistent with the fact that more than half of our patient cohort made insurance claims for injury. Our findings indicate that patients making an injury claim are more likely to harass staff. Specifically, patients with disability injury claims are not only more prone to harass clinic staff but also more likely to engage in sexual harassment. Patients with disability injury claims are typically unemployed; while about 25% of our total patient population were unemployed, 50% of those who harassed the staff fell into this category. This over-representation of unemployed harassers may be linked to their psychological issues.

In our study, the majority of patients who harassed staff were either White or South Asian, reflecting the predominance of these ethnicities in the population. This also suggests incidents of racism and discrimination against the pain clinic staff (12, 13, 15, 16). The clinic involved in this study was staffed by three Black and one Asian professional. Harassment of healthcare staff is typically reactive, often directed at convenient targets, and disproportionately affects ethnic minority professionals (12, 13, 16). Due to societal prejudices, pain clinicians from racialized minority or immigrant backgrounds are frequently the prime targets of abuse (12, 16). Such discrimination is traumatic for the affected healthcare professionals (12, 13, 16). There is a critical need for peer support networks to support immigrant and racialized minority professionals who are particularly vulnerable to harassment.

Our study revealed that the majority of patients who harassed staff were females, possibly reflecting the female predominance in the population. Sexual harassment was exclusively perpetrated by female patients, particularly those with disability injury claims. Some patients may perceive sexual harassment as harmless or even flattering, but it is both insulting and unfair to clinic staff. Mitigating sexual harassment can be achieved through patient education, setting clear boundaries, chaperoned care, using electronic patient-clinic communication, issuing warnings to stop harassment, discouraging inappropriate behavior, and redirecting patients who challenge boundaries. Patients who fail to improve may be discharged after they receive adequate notification and prescriptions. All incidents and outcomes should be documented. In our study, all instances of sexual harassment from patients were firmly rejected and addressed. However, four patients were discharged due to persistent sexually harassing behavior.

Our study revealed that four patients made unfounded retaliation complaints against the pain clinician. Retaliation complaints, a type of harassment made by dissatisfied patients, have negative impacts on public safety and societal healthcare. They undermine various beneficial functions of the pain clinic, such as mitigating the opioid misuse crisis, implementing multimodal analgesia, safely prescribing sedatives, reducing emergency services utilization, and facilitating post-trauma rehabilitation.

Harassment of pain clinic staff is problematic partly due to the opioid misuse crisis (8, 9, 11), which has been further complicated by the recent pandemic (9, 10, 18). Socioeconomic challenges, racism, or societal issues may also contribute to the problem of healthcare staff harassment (16, 19, 20). Nonetheless, all harassment incidents in our study were managed appropriately, leading to compassionate, equitable, and ethical outcomes (2, 4, 19, 21). Most incidents were resolved through tactful de-escalation, interactive communication, patient education, and counseling.

Despite the challenges posed by chronic pain patients, they should be treated with compassion, equity, and fairness. Patients should be reminded of the boundaries of appropriate behavior and the importance of treating healthcare professionals with decency. Psychotherapy and counseling should be provided for suitable patients. Those who refuse to behave appropriately should be referred to another pain service better equipped to handle them, with the referral letter indicating the harassment issue. Some difficult patients may be effectively managed through telehealth consultations and occasional in-person clinic visits (22, 23). When pain clinic staff face harassment, the best response is to remain compassionate, consistent, and committed.

The prospective cohort methodology significantly reduced the risk of selection bias, information recall bias, and outcome bias. It enabled the collection of data on multiple variables and outcomes over specific periods, facilitating the discovery of new associations between variables and outcomes. While the consecutive sampling method reduced bias, it necessitated a prolonged study duration. However, this study was limited to a single pain clinic. Further research should encompass multiple pain clinics to enhance generalizability. The study protocol was registered on the Clinical Trials PRS (Protocol Registration and Results System) website, with the PRS number NCT05876104. The study data will be made available for future research via secure networks. Informed consent was obtained from all patients, and there is no conflict of interest among the authors.

The sociomedical problem of pain clinic staff harassment is significant, partly due to opioid misuse, the pandemic, racism, socioeconomic factors, and other societal challenges. This study confirmed that pain clinic staff harassment is primarily caused by patients who are mostly female, unemployed, making injury claims, demanding higher drug doses, and lacking regular healthcare providers. While most chronic pain patients are reasonable, some present challenges. Abusive patients should be provided with anxiolytic therapy, clear behavioral boundaries, counseling, distraction therapy, and empathy. Patients should be treated with compassion, equity, and fairness.

Pain clinics should establish professional networks to support each other against harassment and implement protocols and measures to protect staff from such incidents. Healthcare staff harassment should always be documented, analyzed, and managed properly.

## Data Availability

The dataset presented in the study is available on request from the corresponding author during submission or after publication.

## Clinical Trial Registration Code:

NCT05876104.
